# Detection of cytokeratins 19/20 and guanylyl cyclase C in peripheral blood of colorectal cancer patients

**DOI:** 10.1038/sj.bjc.6990289

**Published:** 1999-04

**Authors:** S A Bustin, V G Gyselman, N S Williams, S Dorudi

**Affiliations:** Academic Department of Surgery, St Bartholomew's and the Royal London School of Medicine and Dentistry, London, UK

**Keywords:** solid tumours, micrometastases, cytokeratins, guanylyl cyclase C, RT-PCR, quantitation

## Abstract

The clinical significance of detecting supposed tumour cell-derived mRNA transcripts in blood using the polymerase chain reaction (PCR) remains unclear. We have used a fully quantitative 5′-nuclease RT-PCR assay to screen for the expression of cytokeratins (ck) 19 and 20 and guanylyl cyclase C (GCC) in the peripheral blood of 21 healthy controls and 27 colorectal cancer patients. Expression of cytokeratin 19 and 20 mRNA was detected in 30% and 100% of samples, respectively, taken from healthy volunteers. There was no apparent difference in ck19 and ck20 mRNA transcription levels between controls and patients, or between patients with different Dukes' stages. While GCC mRNA was detected in only 1/21 control samples, it was expressed in approximately 80% of patients, although again there was no correlation between GCC levels and disease stage. Transcription levels of all three markers varied considerably between samples, even between samples taken from the same person at different times. We conclude that neither ck19 nor ck20 are reliable markers for the detection of colon epithelial cells in peripheral blood and that an evaluation of the usefulness of GCC awaits further longitudinal studies. © 1999 Cancer Research Campaign


*British Journal of Cancer* (1999) **79**(11/12), 1813–1820
© 1999 Cancer Research Campaign
Article no. bjoc.1998.0289

### Detection of cytokeratins 19/20 and guanylyl cyclase C in peripheral blood of colorectal cancer patients

**SA Bustin, VG Gyselman, NS Williams and S Dorudi**

Academic Department of Surgery, St Bartholomew's and the Royal London School of Medicine and Dentistry, London, UK

**Summary** The clinical significance of detecting supposed tumour cell-derived mRNA transcripts in blood using the polymerase chain reaction (PCR) remains unclear. We have used a fully quantitative 5′-nuclease RT-PCR assay to screen for the expression of cytokeratins (ck) 19 and 20 and guanylyl cyclase C (GCC) in the peripheral blood of 21 healthy controls and 27 colorectal cancer patients. Expression of cytokeratin 19 and 20 mRNA was detected in 30% and 100% of samples, respectively, taken from healthy volunteers. There was no apparent difference in ck19 and ck20 mRNA transcription levels between controls and patients, or between patients with different Dukes' stages. While GCC mRNA was detected in only 1/21 control samples, it was expressed in approximately 80% of patients, although again there was no correlation between GCC levels and disease stage. Transcription levels of all three markers varied considerably between samples, even between samples taken from the same person at different times. We conclude that neither ck19 nor ck20 are reliable markers for the detection of colon epithelial cells in peripheral blood and that an evaluation of the usefulness of GCC awaits further longitudinal studies.

**Keywords:** solid tumours; micrometastases; cytokeratins; guanylyl cyclase C; RT-PCR; quantitation

Microscopic residual disease is not detectable by current histological methods and such occult micrometastases represent the single most important cause of treatment failure in cancer patients (Deans et al, 1992). Since most circulating tumour cells are destroyed in the systemic circulation (Tarin et al, 1984; Glaves et al, 1988), their identification has required the development of sensitive molecular biology-based methods. The discovery that many tumours release large amounts of their DNA into the blood has led to the application of the polymerase chain reaction (PCR) to the detection of structural abnormalities in tumour cell-derived DNA in blood (Chen et al, 1996; Nawroz et al, 1996). Alternatively, reverse transcription PCR (RT-PCR) has been used to detect tumour tissue-specific gene expression (Johnson et al, 1995). Although such advances appear to have resulted in the molecular detection of systemic tumour cells in patients with many different cancers, the relationship between circulating cells and ultimate prognosis is still unclear (reviewed in Bustin and Dorudi, 1998).

Colorectal cancer lacks a tumour-specific marker and several mRNAs, including carcinoembryonic antigen (Gerhard et al, 1994; Mori et al, 1995; Jonas et al, 1996; Liefers et al, 1998), the lymphatic homing receptor CD44 (Wong et al, 1997) and various cytokeratins (Burchill et al, 1995; Gunn et al, 1996; Denis et al, 1997), have been evaluated as potential markers of occult blood, lymph node and/or bone marrow metastasis. Expression of cytokeratin 19 (ck19; Genbank accession no Y00503) has been reported in a wide range of tissues (Quinlan et al, 1985) including normal lymph nodes, bone marrow and peripheral blood (Burchill et al, 1995; Gunn et al, 1996). Expression of cytokeratin 20 (ck20; Genbank accession no X73501), detected in most colorectal cancers and their metastases (Moll et al, 1992), initially appeared to be more limited (Moll et al, 1993) and its detection by RT-PCR has been used to identify occult metastases in colorectal cancer patients (Denis et al, 1997; Funaki et al, 1997; Dorudi et al, 1998). However, ck20 mRNA transcription (Denis et al, 1997) as well as protein expression (Litle et al, 1997) has been described recently in the blood of healthy controls, making its use in the detection of micrometastatic cells questionable (Wyld et al, 1998). Most recently, expression of guanylyl cyclase C (GCC; Genbank accession no U20230/S57551), which specifies the guanylate cyclase-coupled enterotoxin receptor, has been proposed as a selective marker for metastatic colorectal tumours in extra-intestinal tissues (Carrithers et al, 1996; Waldman et al, 1998).

We have used the fluorogenic 5′-nuclease (*Taq*man) assay (Holland et al, 1991) which, with appropriate instrumentation, allows full quantitation and analysis of the PCR reaction, to investigate the relevance of detecting ck19 and ck20 as well as GCC in the peripheral blood of patients with colorectal cancer. T7 polymerase-transcribed templates specific to each marker were used to generate standard curves that allowed absolute quantitation of mRNA copy numbers. Our results reveal that ck19/20 expression occurs too frequently in the peripheral blood of healthy volunteers to be clinically useful in the assessment of colorectal cancer patients. The biological significance of detection of GCC transcription awaits complete validation in further longitudinal studies.

### MATERIALS AND METHODS

### Patients

This study was approved by the Local Research Ethics Committee of the East London and City Health Authority. Venous blood samples were obtained from 27 patients with colorectal cancer

*Received 24 March 1998*
*Revised 14 August 1998*
*Accepted 21 August 1998*

*Correspondence to:* SA Bustin

Table 1 Clinical and biological status of patients with colorectal carcinoma

| Patient | Sex/age | Duke's stage | ck19 | ck20 | GCC Peri-operative | GCC 1 month post-operative |
|---|---|---|---|---|---|---|
| 527 | F/66 | B | – | + | – | – |
| 528 | M/47 | C2 | + | + | + | + |
| 529 | F/75 | D | – | + | + | + |
| 530 | F/73 | C2 | – | + | – | + |
| 531 | F/78 | B | – | + | + | + |
| 532 | M/75 | D | – | + | – | – |
| 533 | M/67 | B | – | + | + | + |
| 534 | M/61 | B | + | + | + | + |
| 536 | M/62 | C | + | + | – | – |
| 537 | M/56 | D | – | + | – | + |
| 538 | M/66 | B | – | + | – | – |
| 539 | M/71 | D | – | + | – | – |
| 541 | M/49 | D | – | + | + | + |
| 542 | F/39 | C | + | + | + | + |
| 543 | F/80 | B | – | + | + | + |
| 544 | F/43 | B+C[a] | – | + | + | + |
| 545 | M/39 | A | – | + | + | + |
| 546 | M/75 | B | + | + | + | + |
| 547 | M/67 | B | + | + | + | + |
| 548 | M/69 | D | – | + | + | + |
| 549 | F/62 | B | + | + | + | + |
| 550 | M/62 | C | – | + | + | + |
| 551 | F/68 | B | – | + | + | + |
| 552 | F/84 | B | – | + | + | + |
| 553 | F/55 | A | – | + | + | + |
| 554 | F/44 | B | – | + | + | + |
| 555 | F/76 | B | – | + | + | + |

[a]Synchronous tumour.

(age 39–80 years) at the time of operation. A second sample was obtained from 15/27 patients at the time of their 1-month follow-up visits. Patient data are summarized in Table 1. In each case 3-ml samples of peripheral blood were obtained and stored in sterile lithium heparin vacutainers for a maximum of 2 h before RNA was extracted. In our hands use of heparinized containers, combined with the RNA extraction protocol detailed below, did not inhibit the subsequent RT-PCR reaction. Blood samples were taken from 21 healthy control subjects (age 26–61 years). Additional samples were taken from 14/21 at weekly intervals for 3 weeks.

### Tumours

To confirm expression of the three potential marker genes in colorectal tumours, two separate areas from each of five tumours (four patients, two synchronous tumours) were removed, washed in distilled water, blotted dry and weighed.

### RNA extraction

All RNA extractions were carried out in a separate room in a class 2 containment hood using dedicated pipettes with aerosol-resistant tips (Gilson). For each 3-ml blood sample, total RNA was extracted in $2 \times 1.5$ ml aliquots using the RNeasy Blood Mini Kit (Qiagen Ltd, Crawley, UK). Manufacturer's instructions were followed except that following lysis of red blood cells, leucocytes were given two additional washes with 3-ml EL (Qiagen Ltd) buffer. This was to ensure complete removal of any potential RT-PCR inhibitors. RNA was eluted in distilled DEPC-treated water, the two aliquots were combined and stored at – 70°C at a concentration of 75 ng µl. RNeasy Mini Kits (Qiagen Ltd) were also used to extract approximately 25 µg of total RNA from $10^6$ T84 cells, a human colorectal cancer cell line. This RNA was stored at – 70°C at a concentration of 50 ng µl. RNA from tumour tissue was extracted using RNeasy Mini kits (Qiagen, Crawley, UK) according to manufacturer's instructions and stored at – 70°C at a concentration of 50 ng µl. All quantitation was carried out using a Hitachi U-3000 spectrophotometer.

### Primers and probes

All probes were labelled with a fluorescent dye (6-carboxy-fluorescein, FAM) and a quencher (6-carboxy-tertramethyl-rhodamine, TAMRA) and were purchased, together with their respective primer sets from PE Applied Biosystems, Warrington, UK. Cytokeratin primers and probes were designed using Primer Express software (PE-ABI) with manual fine-tuning. The ck20 primers bind to sequences in ck20 exons 1 and 2, generating an amplicon of 86 bp, with the probe hybridizing to both exons (Figure 1A). The ck19 primers bind to sequences in exons 2 and 3, generating an amplicon of 75 bp, with the probe binding to both exons (Figure 1B). Two design features minimize amplification and detection of the ck19 pseudogene (Genbank accession no M33101). The downstream primer contains two mismatches with the ck19 pseudogene and the probe contains a further three

 *© Cancer Research Campaign 1999*

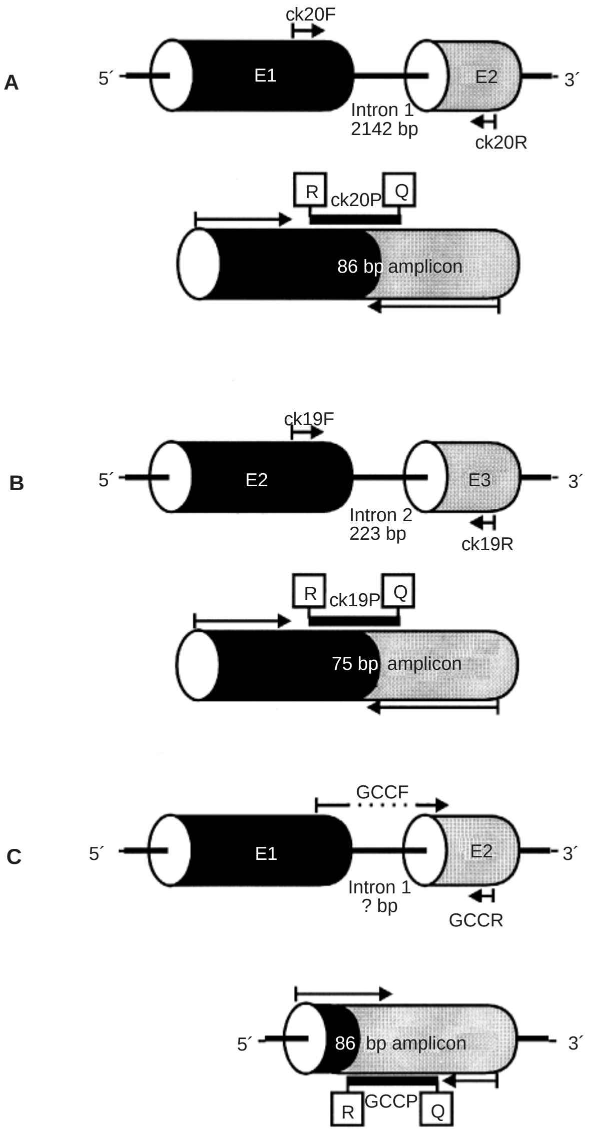

**Figure 1** Location of cytokeratin and GCC primers and probes. E1, E2, E3 designate exons, ck20F, ck19F, GCCF and ck20R, ck19R, GCCR designate forward and reverse primers, respectively, and ck20P, ck19P, GCCP designate the hybridization probe that has the reporter (R) at its 3′-end and the quencher (Q) at its 5′-end

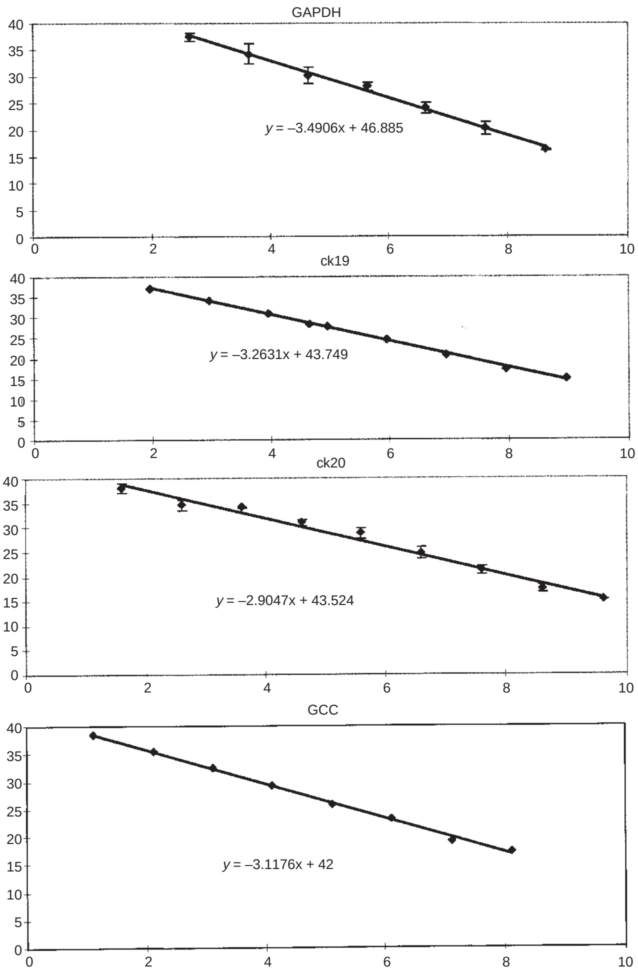

**Figure 2** Standard curves of RT-PCR amplification of GAPDH, ck19, ck20 and GCC amplications. The log starting copy number is plotted against the threshold cycle $C_t$. Error bars indicate standard deviations mismatches. Since the probe is blocked at its 3′-end by the quencher, it cannot be extended and stabilized during the PCR, making it less likely to remain hybridized to the pseudogene. The forward GCC primer binds to sequences in exons 1 and 2 and, with the downstream primer, generates an amplicon of 86 bp (Figure 1C). The glyceraldehyde 3-phosphate dehydrogenase (GAPDH; Genbank accession no J04038) probe/primer set spans introns 2 and 3, generates an amplicon of 226 bp and the probe binds to exon 3. It is identical to the PE-ABI set (402869), except that the probe was synthesized with the same reporter dye (FAM) as the cytokeratin probes. All primer/probe sequences are detailed in Table 2.

### RT-PCR reactions

The one-tube/one enzyme RT-PCR protocol was consistently more sensitive in our hands than separate reverse transcription followed by PCR (Dorudi et al, 1998), and we therefore used this protocol with all 5′-nuclease assays. The volume of each reaction was 25 μl, allowing us to analyse each sample five times. Since each RT-PCR reaction was carried out in duplicate, this resulted in ten quantitations per RT-PCR sample. To prevent carry-over of contaminating amplified DNA, the reaction was carried out in the presence of dUTP. Prior to RT, the RNA template was heated for 2 min at 50°C in the presence of 0.01 U $l^{-1}$ uracil *N*-glycosylase. Following 30 min RT at 60°C and 5 min denaturation at 92°C, PCR was carried out for 40 cycles of 20 s at 92°C and 30 s at 60°C in the presence of an oligonucleotide probe containing a fluorescent reporter at its 5′-end and a quencher at its 3′-end. Following target amplification, the probe anneals to the amplicon and is displaced and cleaved between the reporter and quencher dyes by the nucleolytic activity of the polymerase. The amount of product resulting in detectable fluorescence at any given cycle within the

© Cancer Research Campaign 1999

**Table 2** Primer and probe sequences

| Primer/probe sets | Amplification | Primers | Probe (5´FAM-3´TAMRA) |
|---|---|---|---|
| ck20 | exons 1 and 2 | Forward: GCGACTACAGTGCATATTACAGACAA<br>Reverse: GCAGGACACACCGAGCATTT | TGAGCATCCTTAATCTGACTTCGCAGCTCTTC |
| ck19 | exons 2 and 3 | Forward: TCGACAACGCCCGTCTG<br>Reverse: CcaCGCTCATGCGCAG | CCGAACCAAGTttGAGACGGAaCAGG |
| GCC | exons 1 and 2 | Forward: ACGTCTGCAAAATGCTGGC<br>Reverse: GGCAGTCGCCTGAGTTATGAA | AGACCATCCGAATACATGAAAGTAGCGTTCACAG |
| GAPDH | exons 2 and 3 | Forward: GAAGGTGAAGGTCGGAGTC<br>Reverse: GAAGATGGTGATGGGATTTC | CAAGCTTCCCGTTCTCAGCC |

Oligonucleotide sequences are described in the 5'–3'-direction. Mismatches between ck19 and its pseudogene in the reverse primer and the probe are indicated by the use of lower case letters.

exponential phase of PCR is proportional to the initial number of template copies. The number of PCR cycles (threshold cycle, $C_t$) needed to detect the amplicon is therefore a direct measure of template concentration. Two no-template controls were included with every amplification run; one was prepared prior to opening all the tubes and dispensing the various reagents, the other at the end of the experiment. This allowed us to monitor any contamination arising during the handling of the reagents. Reactions were recorded and analysed using the ABI 7700 Prism Sequence detection system (Perkin-Elmer Applied Biosystems, Warrington, UK).

### Generation of standard curves

Accurate quantitation of each potential marker requires that the absolute quantities of each transcript are known. Amplicons specifying GAPDH, ck19, ck20 and GCC were amplified from T84 mRNA using the appropriate primers and cloned behind the T7 RNA polymerase promoter in the pCR2.1 TA-cloning vector (Invitrogen, Leek, The Netherlands). After linearization of the plasmid, RNAs were transcribed using T7 RNA polymerase (Promega) to generate transcripts of 235, 195, 184 and 195 b respectively. Contaminating DNA was removed by DNAse digestion (Boehringer Mannheim). RNAs were purified using a cleanup kit (Qiagen) and quantitated on a Genequant spectrophotometer (Pharmacia, St Albans). A total of 1 μg of an average 1000 b mRNA contains $1.9 \times 10^{12}$ molecules. Serial dilutions of the T7 transcripts were carried out in duplicate from $1 \times 10^9$ molecules down to ten molecules and used in RT-PCR reactions. Reactions were carried out four times, each one in triplicate. By plotting the log [calculated copy number] against the threshold cycle, a standard curve was obtained for each amplicon. The copy numbers (N) of unknown samples were calculated from the regression lines according to the formula: $N = Ct - b/m$, where $C_t$ is the threshold cycle, b is the y-intercept and m is the slope of the standard curve lines.

### RESULTS

### Standard curves

The standard curves in Figure 2 show consistent amplification over seven orders of magnitude for all four targets, resulting in the detection of fewer than 100 copies of mRNA for ck19/20 and GCC. The minimum number of GAPDH mRNA molecules detected was 400. Differences are likely to be caused by differing amplification efficiencies of the four amplicons. The GCC primer/probe combination consistently gave the highest levels of sensitivity, followed by ck19 and 20.

**Table 3** Expression of marker genes in blood, colorectal tumour tissue and tissue culture cells

| Marker | Control blood[a] | Patient blood[a] | Tumour[b] | T84 cell[c] |
|---|---|---|---|---|
| GAPDH ± SD | $4.4 \times 10^9 \pm 3 \times 10^9$ | $5.8 \times 10^9 \pm 7.7 \times 10^9$ | $8.3 \times 10^4 \pm 1 \times 10^6$ | $1.2 \times 10^5 \pm 1.4 \times 10^4$ |
| ck19 ± SD | $2 \times 10^4 \pm 3.2 \times 10^5$ | $9.4 \times 10^3 \pm 2.2 \times 10^4$ | $7.1 \times 10^4 \pm 1.8 \times 10^5$ | $8.2 \times 10^3 \pm 6.7 \times 10^3$ |
| ck20 ± SD | $6.8 \times 10^3 \pm 1.7 \times 10^5$ | $2.9 \times 10^3 \pm 1.9 \times 10^5$ | $3.2 \times 10^4 \pm 8.2 \times 10^5$ | $1 \times 10^4 \pm 1.6 \times 10^3$ |
| GCC ± SD | $0 \pm 1.6 \times 10^4$[d] | $4.6 \times 10^2 \pm 1.1 \times 10^7$[e] | $2.2 \times 10^3 \pm 8.1 \times 10^3$ | $2.5 \times 10^2 \pm 7.9 \times 10^1$ |

The median mRNA copy numbers are expressed [a]per ml of blood, [b]per μg of tissue or [c]per cell. The median number of nucleated blood cells (NBC) in individual blood samples was $3.4 \times 10^6 \pm 1.7 \times 10^6$. [d]The large standard deviation is due to the one healthy volunteer expressing high copy numbers of GCC. [e]The large standard deviation is due to one patient expressing $7 \times 10^7$ copies of GCC per ml blood. Without that patient, the standard deviation would be $8.2 \times 10^2$.

 © Cancer Research Campaign 1999

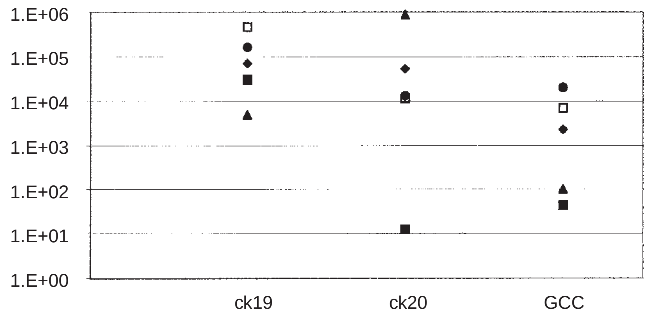

**Figure 3** Scatterplot showing the variation in mRNA transcription (plotted as log copy numbers) between five tumours. Copy numbers are expressed per µg of wet tissue. A comparison within each of the five tumours showed very little variation

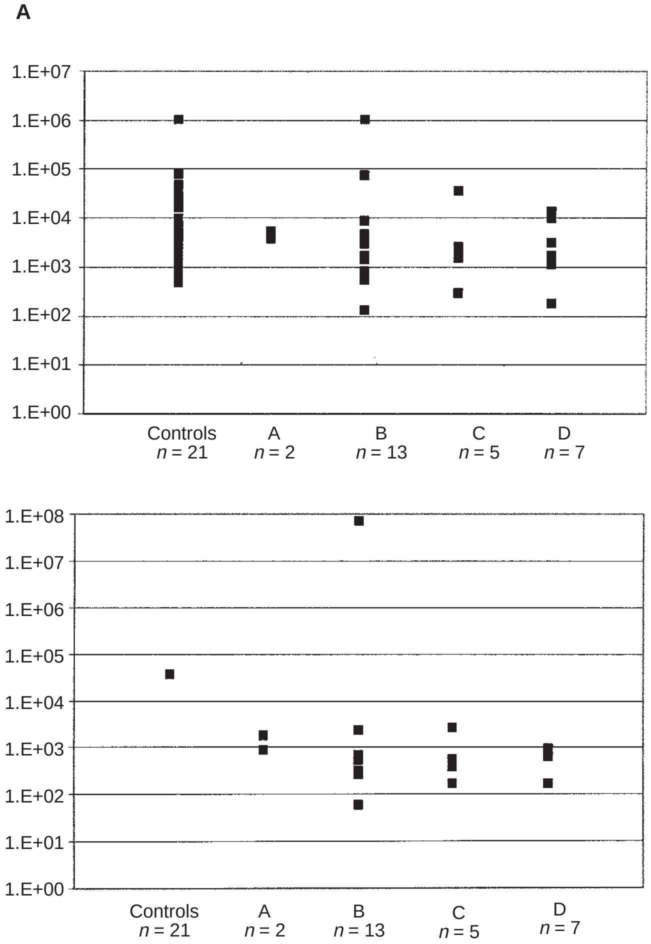

**Figure 4** Lack of correlation between mRNA copy numbers and disease stage in peri-operative peripheral blood samples. The range of mRNA copy numbers obtained with peripheral blood samples from healthy volunteers shows no difference compared with the range obtained in colorectal cancer patients. The scatter chart plots log copy number per ml blood. Similar results were obtained when the copy numbers per cell were plotted. (**A**) ck20; (**B**) GCC. Twenty of the controls and four of the Duke's D patients did not have detectable GCC expression

### ck19/20 and GCC expression in tumour tissue

To quantitate transcription levels in colon epithelial cells, RNA was prepared from five tumours, two of which were synchronous. mRNA for all three markers was detected and median expression levels are shown in Table 3. However, mRNA copy numbers differed widely between different tumours, ranging $4.9 \times 10^3$ to $4.5 \times 10^5$, $6.1 \times 10^1$ to $2.3 \times 10^6$ and $4.6 \times 10^1$ to $1.9 \times 10^4$ respectively for ck19, ck20 and GCC (Figure 3). There was little variation between different sections of the same tumour (results not shown).

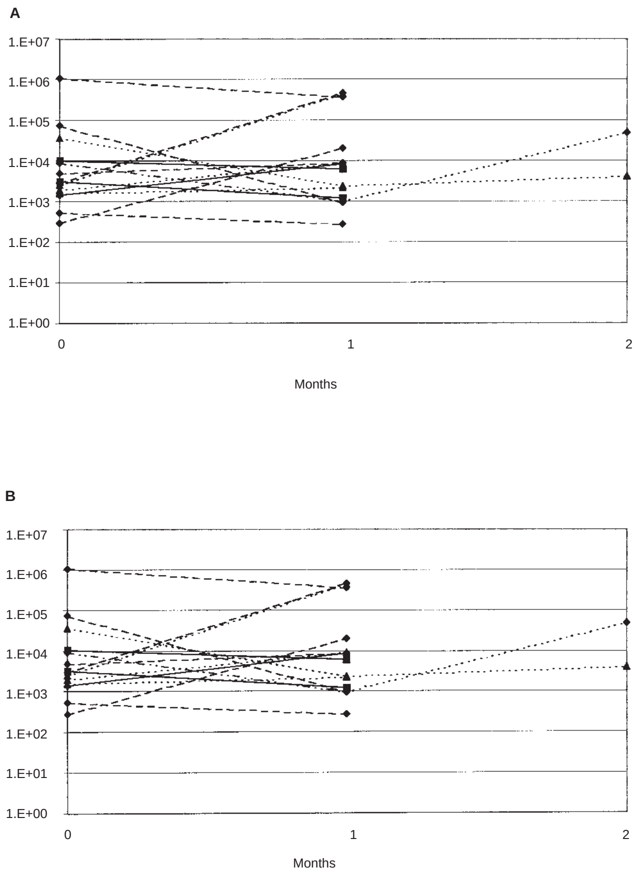

**Figure 5** Post-operative expression of ck20 and GCC in peripheral blood of colorectal cancer patients. The plots track changes in the log copy numbers seen from the time of operation. (**A**) ck20; (**B**) GCC. ◆- - - - Duke's B, ▲- - - - Duke's C, ■—Duke's D

### ck19/20 and GCC expression in blood from healthy volunteers

All 21 healthy controls expressed ck20 and six expressed ck19 mRNA. Again there was significant variation in mRNA copy numbers, which ranged $8.4 \times 10^5$ to $7.3 \times 10^6$ and $3.8 \times 10^2$ to $8.8 \times 10^5$ for ck19 and ck20. Similar variation was also observed in blood samples taken from the same 14 individuals over a 3-week period. Transcription of GCC mRNA was detected in one healthy control. This was confirmed in two additional samples taken at weekly intervals. All three samples also had high levels of ck19 and ck20 mRNA. The copy number of the housekeeping gene GAPDH also showed significant variation, ranging $2 \times 10^2$ to $6.5 \times 10^3$ copies per nucleated blood cell. Median expression levels are shown in Table 3.

### ck19/20 and GCC expression in blood from colorectal cancer patients ck20 mRNA could be detected in all 27 peri-operative blood samples. Median expression levels are shown in Table 3. There was no significant difference in ck20 copy numbers compared to those obtained in healthy controls. There was also no difference between patients with different stages of colorectal cancer (Figure 4). ck19 mRNA was detected in seven blood samples, but there was no correlation with ck20 expression levels. GCC mRNA was detected in 20/27 samples, but 4/7 samples from Duke's D patients were GCC-negative.

One to 2 months post-operatively, expression of all ck20 mRNAs was still apparent at levels similar to those detected in peri-operative samples (Figure 5A). GCC expression was detected in an additional two patients (one Duke's C2, one Duke's D). Interestingly, all of the patients that were GCC-positive at the time of operation remained so (Figure 5B).

### Comparison of mRNA copy numbers between tissue culture cells and nucleated blood cells

We used several preparations of T84 mRNA to determine the copy numbers of GAPDH, ck19/20 and GCC in this colorectal cancer tissue cell line. In contrast to the results obtained in vivo, there was significantly less variation in copy numbers (Table 3). However, GAPDH copy numbers per cell were two orders of magnitude greater than those calculated per nucleated blood cell ($1.9 \times 10^3 \pm 1.2 \times 10^3$). This is relevant, since blood spiking experiments with tissue culture cells are generally used to assess the sensitivity of the RT-PCR reaction. This result suggests that sensitivity levels obtained this way must be treated with caution.

### DISCUSSION

Although RT-PCR is widely used to identify mRNA presumed to originate from circulating viable tumour, the clinical significance of their detection remains unknown (Bustin and Dorudi, 1998). There are several problems associated with its use as a routine tool in clinical medicine. Standardization of the technique across laboratories and questions concerning sensitivity and specificity are vital issues, which can hinder the translation of results into clinical practice. This is highlighted by our finding that mRNA copy numbers in T84 cells are two orders of magnitude greater than in vivo. Since all previous reports base their claimed sensitivity on spiking experiments that use tissue culture cells, our results suggest that their use overestimates the sensitivity of the RT-PCR assay. The difficulty in obtaining quantitative information has limited the prognostic power of the RT-PCR and, although several methods have been devised to attempt better quantitation (Fukuhara et al, 1992; Hayashi et al, 1995; Leygue et al, 1996), none are easily translated into routine use.

Paradoxically, as protocols have become more refined and detection limits have been reduced, several reports have suggested that tissue-specific genes may be 'illegitimately' transcribed in non-specific cells. Illegitimate transcription was originally defined as the low-level transcription of any gene in any cell and was proposed to account for the detection of mRNA transcripts of several tissue-specific genes in human non-specific cells (Chelly et al, 1989; Berg et al, 1990). Transcription is initiated from the normal promoter (Chelly et al, 1991) and, although the effect has been validated for a large number of mRNAs (Cooper et al, 1994), it is not observed for every mRNA (Sorg et al, 1991). There are conflicting reports both as to whether illegitimate transcription of cytokeratins contributes to the detection of false positives (Burchill et al, 1995; Foss et al, 1995; Denis et al, 1997; Dingemans et al, 1997; Hanekom et al, 1997; Zippelius et al, 1997) and which cytokeratins are affected (Traweek et al, 1993).

Our results shed some light on this. They confirm previous reports suggesting that haematopoietic cells extracted from healthy volunteers express both ck19 (Burchill et al, 1995) and ck20 mRNA (Denis et al, 1997). Our results differ, in that transcription of ck20 mRNA was detected in all 21 healthy control samples analysed, whereas transcription of the ck19 gene could be detected in only 30% of healthy controls. Expression levels of both are sufficient to generate false positive results and strongly suggest that their usefulness in the detection of micrometastatic tumour cells by conventional RT-PCR is limited. Of course, ck19/20 transcripts may originate from non-tumour epithelial cells present in the circulation and the sporadic nature of their presence may account for the differing levels of transcription we have detected. The ability to quantitate expression levels should permit a more accurate assessment of transcription above background levels.

We have detected GCC mRNA in only one normal healthy control so far. This was confirmed in two further samples, taken at 1-week intervals. GCC copy numbers in these samples were higher than copy numbers in 26/27 of the patients. Interestingly, these samples also contained very high copy numbers of both ck19 and 20 mRNA. The meaning of these results remains unknown and may reflect unusually high levels of illegitimate transcription for all three genes in that person, or it could indicate the presence of epithelial cells in the peripheral blood.

To determine whether quantitative RT-PCR can overcome the problems associated with either illegitimate transcription or the presence of epithelial cells in the blood, and to ascertain whether detection of ck19/ck20 and GCC mRNA might correlate with disease stage, we applied our protocol to the screening of blood from patients undergoing surgery for colorectal cancer. As is evident from Figure 4A, their pre-operative blood samples showed no significant difference in the levels of ck20 mRNA compared with healthy control subjects, and the same was true for ck19. There also appeared to be no difference in ck19/20 copy numbers between patients with lymph node-negative tumours, those with Duke's stage C and those with confirmed distant metastatic spread to the liver. Figure 5A shows that the same is true after 1 month's follow-up. This is in agreement with a recent report which suggests that there is no correlation between the detection of ck20 mRNA in the peripheral blood and disease progression (Wyld et al, 1998). Expression of GCC, on the other hand, which had been undetectable in 20/21 controls, was identified in 20/27 pre-operative samples (Figure 4B). Since GCC is a tissue-specific, not a tumour-specific, marker this suggests that the RT-PCR is detecting the presence of colon epithelial cells in patients' blood. There was no correlation between disease stage and GCC copy number in pre-operative blood samples, in contrast to a previous report that suggests GCC transcription can be detected only in the peripheral blood of Duke's C and D patients (Carrithers et al, 1996). Our data also suggest that all patients that tested positive for GCC expression in their peri-operative samples still have colon epithelial cells circulating in their blood 1 month after surgery and identify two patients (one Duke's C2, one Duke's D) in whose blood GCC expression can now be detected (Figure 5B).

 *© Cancer Research Campaign 1999*

In conclusion, we have used a real-time RT-PCR assay to show that healthy volunteers express both ck19 and ck20 mRNA in their peripheral blood. This raises serious doubt about their usefulness in colorectal cancer. This doubt is reinforced by the significant variation in their copy numbers. We have been unable to show a correlation between ck19 and ck20 transcription and disease stage in peri-operative peripheral blood samples of colorectal cancer patients. One to 2 months after surgery, there are still epithelial cells detectable in blood samples from every patient, regardless of Duke's stage. The same is true for the 74% of patients that showed expression of GCC in their peri-operative blood samples. The ability to quantitate is invaluable in distinguishing background levels of transcription and it will be crucial to validate this technique in prospective studies. Finally, it must always be remembered that, by themselves, such novel techniques will not translate into improved patient outcome unless corresponding advances are made in adjuvant treatment modalities.

### ACKNOWLEDGEMENTS

This work was supported by grants from the London Immunotherapy Cancer Trust and from the Special Trustees of the Royal London Hospital.

### REFERENCES

Berg LP, Wieland K, Millar DS, Schlosser M, Wagner M, Kakkar VV, Reiss J and Cooper DN (1990) Detection of a novel point mutation causing haemophilia A by PCR/direct sequencing of ectopically-transcribed factor VIII mRNA. *Hum Genet* **85**: 655–658

Burchill SA, Bradbury MF, Pittman K, Southgate J, Smith B and Selby P (1995) Detection of epithelial cancer cells in peripheral blood by reverse transcriptase-polymerase chain reaction. *Br J Cancer* **71**: 278–281

Bustin SA and Dorudi S (1998) Molecular assessment of tumour stage and disease recurrence using PCR-based assays. *Mol Med Today* **4**: 389–396

Carrithers SL, Barber MT, Biswas S, Parkinson SJ, Park PK, Goldstein SD and Waldman SA (1996) Guanylyl cyclase C is a selective marker for metastatic colorectal tumors in human extraintestinal tissues. *Proc Natl Acad Sci USA* **93**: 14827–14832

Chelly J, Concordet JP, Kaplan JC and Kahn A (1989) Illegitimate transcription: transcription of any gene in any cell type. *Proc Natl Acad Sci USA* **86**: 2617–2621

Chelly J, Hugnot JP, Concordet JP, Kaplan JC and Kahn A (1991) Illegitimate (or ectopic) transcription proceeds through the usual promoters. *Biochem Biophys Res Commun* **178**: 553–557

Chen XQ, Stroun M, Magnenat JL, Nicod LP, Kurt AM, Lyautey J, Lederrey C and Anker P (1996) Microsatellite alterations in plasma DNA of small cell lung cancer patients. *Nature Med* **2**: 972–974

Cooper DN, Berg LP, Kakkar VV and Reiss J (1994) Ectopic (illegitimate) transcription: new possibilities for the analysis and diagnosis of human genetic disease. *Ann Med* **26**: 9–14

Deans GT, Parks TG, Rowlands BJ and Spence RA (1992) Prognostic factors in colorectal cancer. *Br J Surg* **79**: 608–613

Denis MG, Lipart C, Leborgne J, Lehur PA, Galmiche JP, Denis M, Ruud E, Truchaud A and Lustenberger P (1997) Detection of disseminated tumor cells in peripheral blood of colorectal cancer patients. *Int J Cancer* **74**: 540–544

Dingemans AM, Brakenhoff RH, Postmus PE and Giaccone G (1997) Detection of cytokeratin-19 transcripts by reverse transcriptase-polymerase chain reaction in lung cancer cell lines and blood of lung cancer patients [see comments]. *Lab Invest* **77**: 213–220

Dorudi S, Kinrade E, Marshall NC, Feakins R, Williams NS and Bustin SA (1998) Genetic detection of lymph node micrometastases in patients with colorectal cancer. *Br J Surg* **85**: 98–100

Foss AJ, Guille MJ, Occleston NL, Hykin PG, Hungerford JL and Lightman S (1995) The detection of melanoma cells in peripheral blood by reverse transcription-polymerase chain reaction. *Br J Cancer* **72**: 155–159

Fukuhara T, Hooper WC, Baylin SB, Benson J, Pruckler J, Olson AC, Evatt BL and Vogler WR (1992) Use of the polymerase chain reaction to detect hypermethylation in the calcitonin gene. A new, sensitive approach to monitor tumor cells in acute myelogenous leukemia. *Leuk Res* **16**: 1031–1040

Funaki NO, Tanaka J, Itami A, Kasamatsu T, Ohshio G, Onodera H, Monden K, Okino T and Imamura M (1997) Detection of colorectal carcinoma cells in circulating peripheral blood by reverse transcription-polymerase chain reaction targeting cytokeratin-20 mRNA. *Life Sci* **60**: 643–652

Gerhard M, Juhl H, Kalthoff H, Schreiber HW, Wagener C and Neumaier M (1994) Specific detection of carcinoembryonic antigen-expressing tumor cells in bone marrow aspirates by polymerase chain reaction. *J Clin Oncol* **12**: 725–729

Glaves D, Huben RP and Weiss L (1988) Haematogenous dissemination of cells from human renal adenocarcinomas. *Br J Cancer* **57**: 32–35

Gunn J, McCall JL, Yun K and Wright PA (1996) Detection of micrometastases in colorectal cancer patients by K19 and K20 reverse-transcription polymerase chain reaction. *Lab Invest* **75**: 611–616

Hanekom GS, Johnson CA and Kidson SH (1997) An improved and combined reverse transcription-polymerase chain reaction assay for reliable detection of metastatic melanoma cells in peripheral blood. *Melanoma Res* **7**: 111–116

Hayashi N, Ito I, Yanagisawa A, Kato Y, Nakamori S, Imaoka S, Watanabe H, Ogawa M and Nakamura Y (1995) Genetic diagnosis of lymph-node metastasis in colorectal cancer. *Lancet* **345**: 1257–1259

Holland PM, Abramson RD, Watson R and Gelfand DH (1991) Detection of specific polymerase chain reaction product by utilizing the 5′- - - - -3′ exonuclease activity of *Thermus aquaticus* DNA polymerase. *Proc Natl Acad Sci USA* **88**: 7276–7280

Johnson PW, Burchill SA and Selby PJ (1995) The molecular detection of circulating tumour cells. *Br J Cancer* **72**: 268–276

Jonas S, Windeatt S, O-Boateng A, Fordy C and Allen-Mersh TG (1996) Identification of carcinoembryonic antigen-producing cells circulating in the blood of patients with colorectal carcinoma by reverse transcriptase polymerase chain reaction. *Gut* **39**: 717–721

Leygue E, Murphy L, Kuttenn F and Watson P (1996) Triple primer polymerase chain reaction. A new way to quantify truncated mRNA expression. *Am J Pathol* **148**: 1097–1103

Liefers G-J, Cleton-Jansen A-M, van de Velde CJH, Hermans J, van Krieken JHJM, Cornelisse CJ and Tollenaar RAEM (1998) Micrometastases and survival in stage II colorectal cancer. *N Engl J Med* **339**: 223–228

Litle VR, Warren RS, Moore D and Pallavicini MG (1997) Molecular cytogenetic analysis of cytokeratin 20-labeled cells in primary tumors and bone marrow aspirates from colorectal carcinoma patients. *Cancer* **79**: 1664–1670

Moll R, Lowe A, Laufer J and Franke WW (1992) Cytokeratin 20 in human carcinomas. A new histodiagnostic marker detected by monoclonal antibodies. *Am J Pathol* **140**: 427–447

Moll R, Zimbelmann R, Goldschmidt MD, Keith M, Laufer J, Kasper M, Koch J and Franke WW (1993) The human gene encoding cytokeratin 20 and its expression during fetal development and in gastrointestinal carcinomas. *Differentiation* **53**: 75–93

Mori M, Mimori K, Inoue H, Barnard GF, Tsuji K, Nanbara S, Ueo H and Akiyoshi T (1995) Detection of cancer micrometastases in lymph nodes by reverse transcriptase-polymerase chain reaction. *Cancer Res* **55**: 3417–3420

Nawroz H, Koch W, Anker P, Stroun M and Sidransky D (1996) Microsatellite alterations in serum DNA of head and neck cancer patients. *Nature Med* **2**: 1035–1037

Quinlan RA, Schiller DL, Hatzfeld M, Achtstatter T, Moll R, Jorcano JL, Magin TM and Franke WW (1985) Patterns of expression and organization of cytokeratin intermediate filaments. *Ann N Y Acad Sci* **455**: 282–306

Sorg R, Enczmann J, Sorg U, Heermeier K, Schneider EM and Wernet P (1991) Rapid and sensitive mRNA phenotyping for interleukins (IL-1 to IL-6) and colony-stimulating factors (G-CSF, M-CSF, and GM-CSF) by reverse transcription and subsequent polymerase chain reaction. *Exp Hematol* **19**: 882–887

Tarin D, Price JE, Kettlewell MG, Souter RG, Vass AC and Crossley B (1984) Mechanisms of human tumor metastasis studied in patients with peritoneovenous shunts. *Cancer Res* **44**: 3584–3592

Traweek ST, Liu J and Battifora H (1993) Keratin gene expression in non-epithelial tissues. Detection with polymerase chain reaction. *Am J Pathol* **142**: 1111–1118

Waldman SA, Cagir B, Rakinic J, Fry RD, Goldstein SD, Isenberg G, Barber M, Biswas S, Minimo C, Palazzo J, Park PK and Weinberg D (1998) Use of guanylyl cyclase C for detecting micrometastases in lymph nodes of patients with colon cancer. *Dis Colon Rectum* **41**: 310–315

Wong LS, Cantrill JE, Odogwu S, Morris AG and Fraser IA (1997) Detection of circulating tumour cells and nodal metastasis by reverse transcriptase polymerase chain reaction technique. *Br J Surg* **84**: 834–839

*© Cancer Research Campaign 1999*

Wyld DK, Selby P, Perren TJ, Jonas SJ, Allen-Mersh TG, Wheeldon J and Burchill SA (1998) Detection of colorectal cancer cells in peripheral blood by reverse-transcriptase polymerase chain reaction for cytokeratin 20. *Int J Cancer* **79**: 288–293

Zippelius A, Kufer P, Honold G, Kollermann MW, Oberneder R, Schlimok G, Riethmuller G and Pantel K (1997) Limitations of reverse transcriptase polymerase chain reaction analyses for detection of micrometastatic epithelial cancer cells in bone marrow. *J Clin Oncol* **15**: 2701–2708

*© Cancer Research Campaign 1999*
